# How to design a national genomic project—a systematic review of active projects

**DOI:** 10.1186/s40246-021-00315-6

**Published:** 2021-03-24

**Authors:** Anja Kovanda, Ana Nyasha Zimani, Borut Peterlin

**Affiliations:** Clinical Institute of Genomic Medicine, University Medical Centre Ljubljana, Slajmerjeva 4, Ljubljana, Slovenia

**Keywords:** National genomic projects, Precision medicine, Personalized medicine, Normal genomic variation, Pathological genomic variation, Population genomics, Exposome

## Abstract

An increasing number of countries are investing efforts to exploit the human genome, in order to improve genetic diagnostics and to pave the way for the integration of precision medicine into health systems. The expected benefits include improved understanding of normal and pathological genomic variation, shorter time-to-diagnosis, cost-effective diagnostics, targeted prevention and treatment, and research advances.

We review the 41 currently active individual national projects concerning their aims and scope, the number and age structure of included subjects, funding, data sharing goals and methods, and linkage with biobanks, medical data, and non-medical data (exposome). The main aims of ongoing projects were to determine normal genomic variation (90%), determine pathological genomic variation (rare disease, complex diseases, cancer, etc.) (71%), improve infrastructure (59%), and enable personalized medicine (37%). Numbers of subjects to be sequenced ranges substantially, from a hundred to over a million, representing in some cases a significant portion of the population. Approximately half of the projects report public funding, with the rest having various mixed or private funding arrangements. 90% of projects report data sharing (public, academic, and/or commercial with various levels of access) and plan on linking genomic data and medical data (78%), existing biobanks (44%), and/or non-medical data (24%) as the basis for enabling personal/precision medicine in the future.

Our results show substantial diversity in the analysed categories of 41 ongoing national projects. The overview of current designs will hopefully inform national initiatives in designing new genomic projects and contribute to standardisation and international collaboration.

**Supplementary Information:**

The online version contains supplementary material available at 10.1186/s40246-021-00315-6.

## Background

Genomic medicine is the use of genetic information to inform medical care or predict the risk of disease and has been importantly influenced by novel technology such as whole-exome sequencing and whole-genome sequencing [1, 2]. This has led to a significant improvement of health systems particularly in the diagnosis of rare genetic disorders and cancer [3–7] as well as in the development of precision medicine, which is the use of diagnostic tools and treatments targeted to the needs of the individual patient based on their genomics, epigenomics, proteomics, metabolomics, lipidomics, and other data such as environmental and lifestyle information [3, 8].

Thirty years ago, in 1990, the Human Genome Project was initiated with the primary goal to obtain a highly accurate sequence of the human genome and to identify its genes [9, 10]. It was followed, in 1998, by the Icelandic deCode Project, the first major attempt to link genomic data with other medical and non-medical data [11], and in 2010 by the UK10K project, a collaboration among several UK public and private institutions, to identify genetic causes of rare diseases [12]. In 2015, the large precision medicine initiatives of the USA and China were started (to be completed within the next decade) [13–16]. In Europe, the initiative “Towards access to at least 1 million sequenced genomes in the EU by 2022” started in 2018 with the aim to share genomic information and best practices among member states [13, 14, 17, 18]. There are high expectations on the benefits of whole genomic sequencing in terms of the development of precision medicine including improved and cost-effective diagnostics, more targeted prevention and treatment. Nevertheless, few of the projected gains have been demonstrated and no standards on designing the national genome projects have been developed so far.

With this systematic review, we aimed to provide an overview of available information on active national genome projects worldwide in terms of identifying common characteristics and differences among them, which could provide a basis for developing best practices and standards for the design of national projects and sharing of national genome resources.

## Materials and methods

**Fig. 1:**
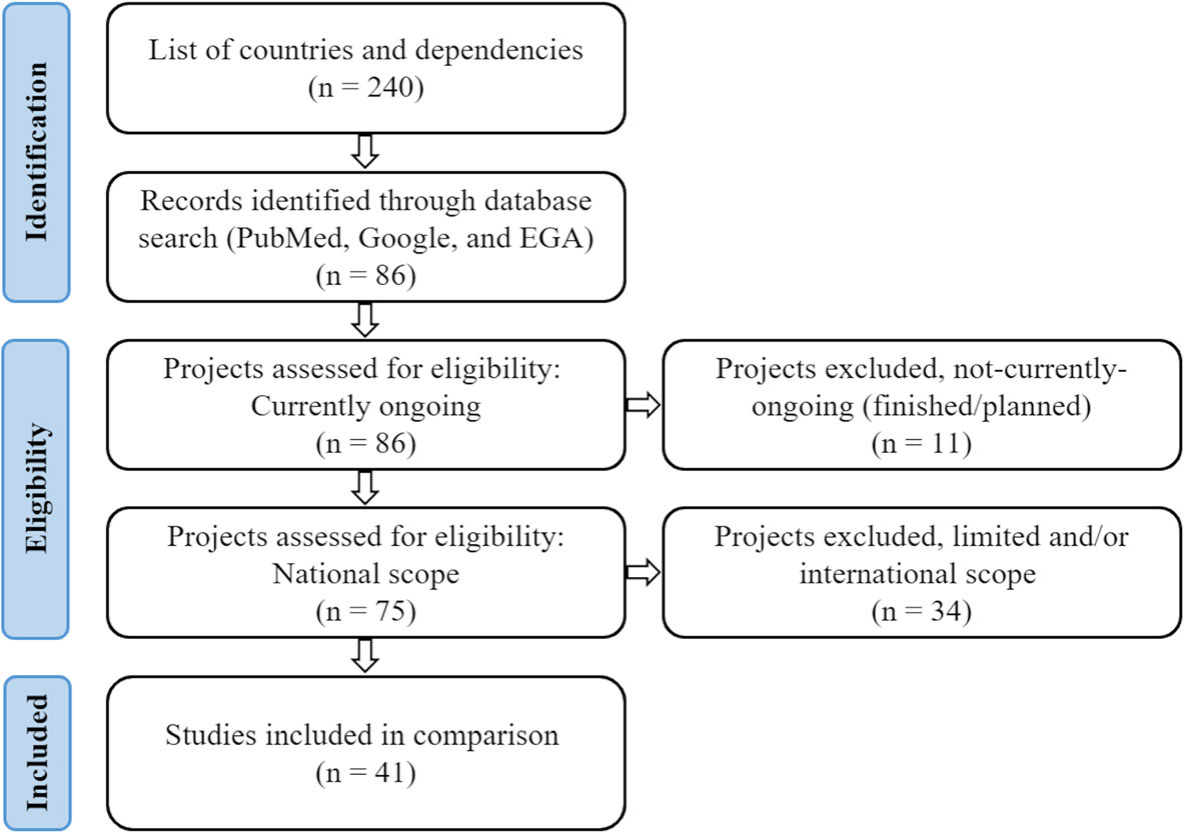
PRISMA type approach to the selection of projects to be included in the analysis

The principles of the PRISMA model were used in the preparation of this work, where possible and appropriate (Fig. 1) [19].

Shortly, to identify existing national genomic projects, PubMed (www.ncbi.nlm.nih.gov/pubmed), Google, and European Genome Phenome Archive-EGA (https://ega-archive.org/) searches were performed in April 2020 by using the search strings: (<country name> [Title]) and (human genome project).

Country names were used in their English language form as listed on Wikipedia countries and dependencies site (https://en.wikipedia.org/wiki/List_of_countries_and_dependencies_by_population).

The following exclusion criteria were used to classify on-going projects: projects concluded prior to the year 2020 or planned with no imminent date in the year 2021 were classified as ‘not currently on-going’; international projects and/or those providing only samples/sequencing facilities were defined as ‘international-scope projects’; and finally, those with unavailable information on key features examined in the article (non-functional websites, announcements with insufficient information, no information in the English language) were defined as ‘limited scope projects'. All three authors analysed and co-reviewed the data and any discrepancies and/or inconsistencies were resolved through agreement. Projects that were not currently on-going, were of limited-scope, and those of international, rather than national scope were excluded from the analysis (Fig. 1).

The complete list of categories for all identified projects is given in Supplement Table 1.

The contents of the individual national project websites were browsed for information pertaining to (1) the aims and scope of the individual project (determining normal and pathological genomic variation, infrastructure (including sequencing and analysis capacities, implementation of standards, data management, education, integration of genomics into existing health-care systems), and intention of facilitating personalized medicine); (2) the number and age structure of included subjects; (3) funding; (4) data sharing goals and methods; and (5) linkage with biobanks, medical data, and non-medical data.

A PRISMA flow-chart diagram was generated using the on-line template (http://www.prisma-statement.org/).

Shared aims of national genomic projects were visualized using an online VENN diagram tool (http://bioinformatics.psb.ugent.be/cgi-bin/liste/Venn/calculate_venn.htpl.).

World maps of national genomic projects were constructed using the online tools available at Mapchart.net (https://mapchart.net/world.html).

## Results

**Fig. 2:**
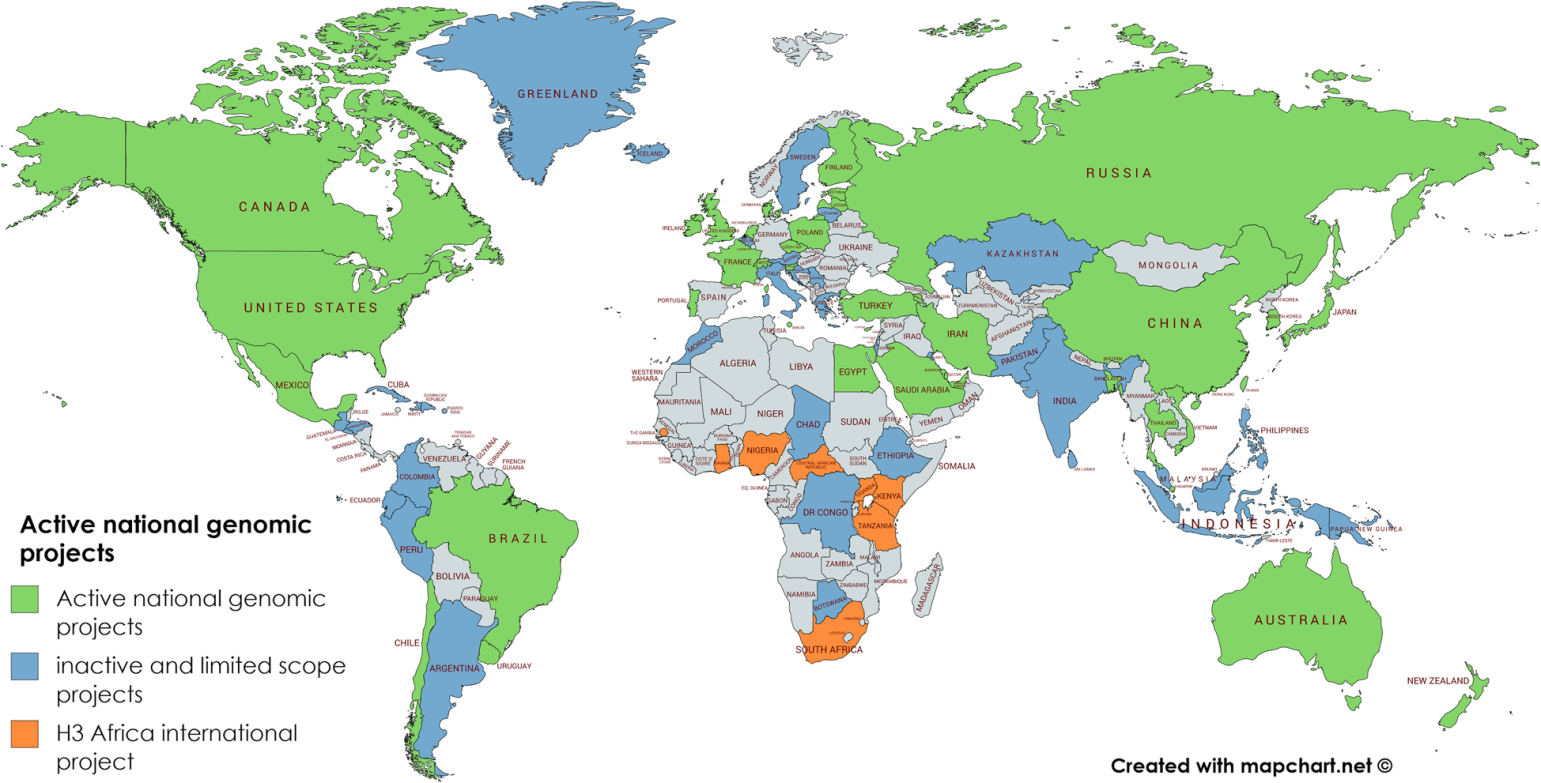
National genomic projects across the world

A total of 86 countries with genomic projects and/or genomic databases were identified among the 240 countries and territories searched, of which 41 projects were currently active, according to the information provided by respective websites [20–60] (Fig. 1). The remaining projects were either not active at the moment or were part of larger international projects (such as H3Africa) and hence not actual ‘national’ projects in a strict sense (Fig. 2). The full list of identified projects is given in Supplement table 1, List of national projects.

### Aims and scope

**Fig. 3:**
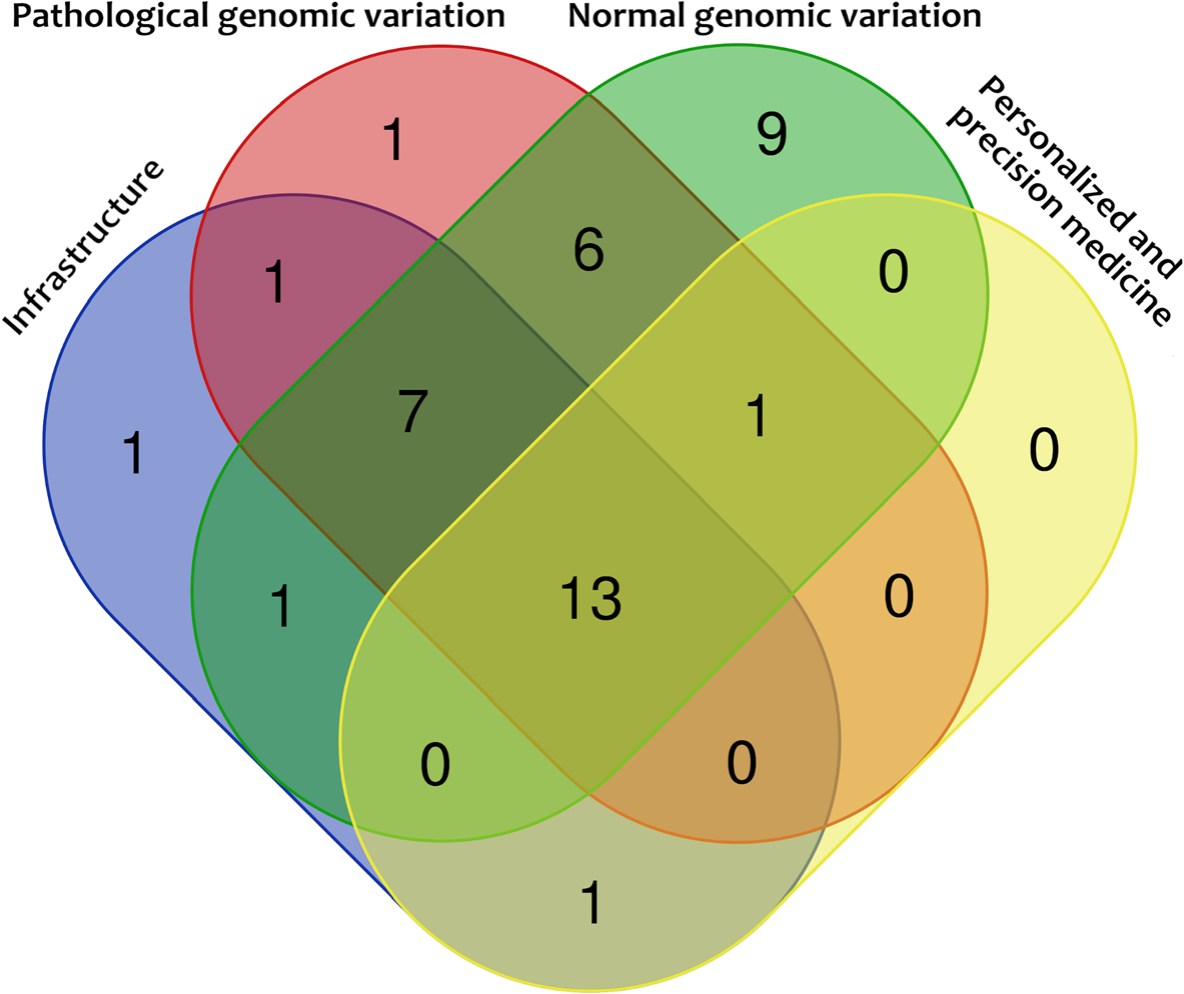
Overlap of major aims of the 41 currently active national genomic projects

The aims of the national genomic projects consisted of four major categories: (1) determining normal genomic variation, (2) determining pathological genomic variation (clinical cohorts such as rare diseases, cancer, complex diseases, etc.), (3) infrastructure, and (4) facilitating personalized and precision medicine (Fig. 3). Additionally, many country-specific aims were also identified, such as history/ethnic studies (Armenia, Brazil, Chile, Hong Kong, Iran, Malta, Mexico, New Zealand, Russia, Singapore, Vietnam) [20–22, 25, 31, 34, 41, 42, 45, 56, 61], drug discovery (Australia, Bahrain, Cyprus, Hong Kong, Japan, Malta, Switzerland, Thailand, UK) [23, 37, 39, 41, 43, 45, 46, 48, 60, 62], reparation efforts (Argentina) [63], or specific health-related goals (infectious diseases interactions—e.g. malaria, tuberculosis in endemic countries) [64, 65].

#### Determining normal genomic variation

The most common aim (90%, 37/41) of national genomic projects was to investigate normal genomic variation by sequencing healthy participants. Because defining health in the context of genomic testing can be challenging, especially in the case of non-penetrant mutations and late-onset disorders, most national projects approached this challenge by either creating cohorts based on demographic data (9/41 projects) and linking them with medical data or specific exclusion criteria, or by specifically identifying healthy individuals (healthy parents from trio testing in rare diseases, longitudinal health-tracking cohorts from previous studies) (Supplement Table 1).

#### Determining pathological genomic variation

The second most common aim was to determine pathological genomic variation through the sequencing of clinical cohorts (71%, 29/41). Seven of the 29 (24%) of the national projects clearly defined the number of subjects they plan to include in their clinical cohorts in advance (France, UK, Australia, Hong Kong, New Zealand, Thailand, and Slovenia), as well as the cohorts or pilot projects themselves. In case of France, 48 clinical cohorts will be included [30], the UK project will include over 190 rare diseases and cancer program [37], and similarly, Australia will include 18 rare disease and cancer flagship projects [66]. The final cohorts in the rest of the projects aiming to determine pathological genomic variation will depend on various factors (funding, pilot initiatives etc.) and will be discussed further below.

#### Infrastructure

The third most common aim, which was reported by roughly two thirds of the projects (59%, 24/41), was the implementation of various infrastructural goals (Supplement Table 1). Infrastructural goals were not a homologous category and reflected the individual projects’ existing sequencing and data-analysis infrastructure, and personnel capacities. The most frequently reported infrastructural project objectives apart from increasing sequencing capacity itself were data management (79%, 19/24), followed by establishing standards of analyses (71%, 17/24), and education (54%, 13/24). Several additional projects (20%, 8/41) intended to approach these goals without reporting them under ‘infrastructure’, probably reflecting cultural conceptual differences in what is considered as infrastructure.

#### Personalized and precision medicine

Finally, 37% (15/41) of the projects presented tangible plans for the development of personalized medicine, although most projects (85%, 35/41) reported personalized medicine as one of their rationales.

As part of the effort toward introducing personalized medicine, a further subset of countries (e.g. Australia, USA, Japan, Switzerland, etc.) intend to use their genomic data for drug discovery/precision therapy (Supplement Table 1).

### Number and age structure of the included subjects

Websites of 37 of 41 national projects (90%) reported information on the total number of subjects to be included in the project. The number of included subjects ranged from a hundred to up to over a million subjects, representing from 0.0001 to 32% of the population. Approximately half of the projects aimed to sequence more than 10,000 subjects, with approximately a quarter aiming to sequence 1000 or less (Table 1). Similarly, in terms of population percentage, only four countries aimed to sequence more than 1% of their population. Of the remaining countries, half aimed to sequence more than 0.02%, and half planned to sequence less than 0.02% of their respective population.

**Table 1:** Numbers of genomes/WES per country and as a percent of the total population

Country	Planned number of WES and/or genome analyses	Country	% of the population to be sequenced
China	100,000,000	Estonia	32.4572
USA	+1,000,000	Ireland	8.1276
Estonia	430,000	China	7.1374
Ireland	400,000	Qatar	3.6400
Japan	250,000	Malta	0.8510
France	235,000	France	0.3503
Canada	130,000	Canada	0.3430
Qatar	100,000	Saudi Arabia	0.3098
Saudi Arabia	100,000	USA	0.3037
Turkey	100,000	Hong Kong	0.2658
UK	100,000	Japan	0.1984
Australia	25,000	Latvia	0.1974
Hong Kong	20,000	Finland	0.1809
Brazil	15,000	Singapore	0.1753
Finland	10,000	UK	0.1505
Taiwan	10,000	Turkey	0.1219
Thailand	10,000	Cyprus	0.1142
South Korea	10,000	Australia	0.0977
Singapore	10,000	Taiwan	0.0424
Mexico	10,000	Denmark	0.0283
Poland	5000	South Korea	0.0193
Malta	4200	Chile	0.0157
Latvia	3769	Thailand	0.0150
Russia	3000	Slovenia	0.0144
Chile	3000	Poland	0.0130
Denmark	1650	New Zealand	0.0121
Czech Republic	1055	United Arab Emirates	0.0102
Cyprus	1000	Czech Republic	0.0099
United Arab Emirates	1000	Mexico	0.0079
Vietnam	1000	Brazil	0.0072
Iran	800	Netherlands	0.0044
Netherlands	750	Uruguay	0.0023
New Zealand	600	Russia	0.0020
Slovenia	300	Vietnam	0.0010
Egypt	110	Iran	0.0010
Bangladesh	100	Egypt	0.0001
Uruguay	80	Bangladesh	0.0001

Of the few projects with missing information on the number of subjects included, most were focused primarily on infrastructure, whereas in the remaining projects the exact number of included subjects was reported to be determined during the project (Supplement Table 1).

The age structure of healthy subjects was reported in five projects. In the projects that provided this information, the most common strategy for determining normal genomic variation was to include the general adult population or existing health-tracking cohorts. In the case of pathological genomic variation, some groups of minors were also planned (e.g. in rare diseases). For detailed information on the included cohorts, please see the ‘Discussion’ section.

### Funding

Approximately half (51%, 21/41) of all national projects stated the total funding planned (Supplement Table 2). The declared amounts reflect the scopes of the individual projects, ranging from 0.32 M USD to over 9200.00 M USD. Roughly half (49%, 20/41) of national genomic projects reported public funding, with some projects having mixed state and federal (Australia) or EU co-funded projects (e.g. Cyprus, Czech Republic) [35, 36, 46, 49, 57, 67]. The remaining national genomic projects either reported mixed public-private type funding (44%, 18/41) (including for example, USA and Switzerland), or fully private funding (7%, 3/41) (Qatar, Ireland, and Vietnam) [13, 25, 30, 31, 33, 40, 50, 55, 62, 68, 69]. The private funding partners were diverse, including sequencing, investment, and insurance companies, as will be reviewed in the discussion.

### Data sharing goals and methods

Data sharing involves the analysis and curation of genomic and associated information obtained during the projects for public, academic, and/or commercial use with various levels of access. It inevitably concerns ethics and legal issues, identifying stakeholders as well as technical aspects and data security. Data sharing represents an important aspect of the national genomic projects, as most reported their main objectives to be determining normal population genomic variation that will enable the use of personalized and precision medicine. 90% (37/41) of the projects reported their intention of sharing the data obtained (Supplement Table 3), and over half of the projects (54%, 22/41) already implemented some form of data sharing. Of the existing data-sharing solutions, the most common format was a database platform with various levels of access for the public, academia, and researchers, whereas the second most common solution consisted of a fully public database containing anonymized or pooled genomic data. For example, Estonia reports it will make their data and DNA available per request and pending approval of the Ethical committee. On the other hand, several of the projects with private funding report they will provide access for approved pharmaceutical/biotechnology companies and research groups (e.g. Ireland, Switzerland, USA).

### Association with biobanks, medical, and non-medical data

**Fig. 4:**
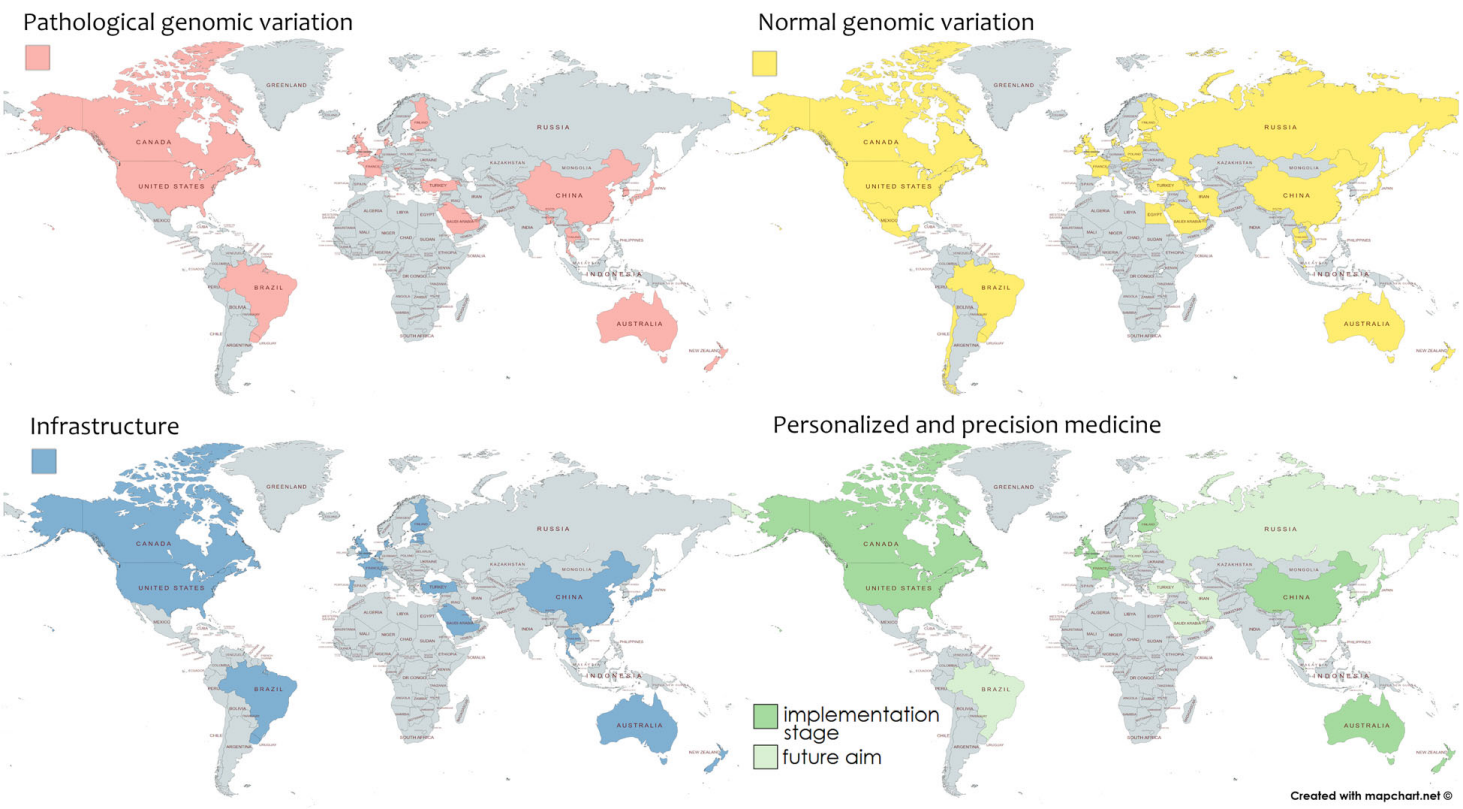
Primary aims of active national genomic projects

The majority of the national projects plan on linking their sequencing data with other medical data (78%, 32/41), existing or planned biobanks (54%, 22/41), and/or non-medical data (24%, 10/41), such as environmental and other factors, as the basis for enabling personal/precision medicine (Table 2) (Fig. 4). Additional countries explicitly plan to establish/connect biobanks and databases during the course of their projects (for example Australia, Slovenia) (Supplement Table 1). Finally, 56% (23/41) projects reported their intention to unify or establish standards for analysis and thus make provisions for adequate data management, two key prerequisites for establishing personalized medicine.

**Table 2:** List of biobanks associated with national genomic projects

Country	Biobank website
Australia	Planned
Bahrain	https://www.moh.gov.bh/GenomeProject?lang=en
Canada	http://tcag.ca/facilities/biobanking.html
China	https://bigd.big.ac.cn/biosample/
Cyprus	https://biobank.cy/the-repository/
Denmark	https://www.danishnationalbiobank.com/access
Estonia	https://genomics.ut.ee/en/access-biobank
Finland	https://www.biopankki.fi/en/finnish-biobanks/
Hong Kong	Planned
Japan	https://www.amed.go.jp/en/program/index04.html
Latvia	http://biomed.lu.lv/en/about-us/related-organisations/lgdb/
Malta	https://www.um.edu.mt/biobank
Mexico	https://mxbiobankproject.org/
Netherlands	https://www.bbmri.nl/
New Zealand	Planned
Qatar	https://www.qatarbiobank.org.qa/search/search?q=database
Russia	https://researchpark.spbu.ru/en/biobank-eng
Slovenia	Planned
Switzerland	https://swissbiobanking.ch/
Turkey	https://bbmri.ibg.edu.tr/
UK	https://www.ukbiobank.ac.uk/
USA	https://allofus.nih.gov/funding-and-program-partners/biobank

## Discussion

Our results show several common goals but also substantial diversity of 41 ongoing national projects across the analysed categories.

### Aims and scope

Since its onset, one of the main aims of genomics has been to enable personalized and precision medicine, which is the use of diagnostic tools and treatments tailored to the needs of the individual patient [3, 8].

Pioneering projects, such as that of the UK, that has been previously reviewed [16, 70]), have focused on determining both the normal and pathological genomic variation (clinical cohorts consisting of rare disease and cancer patient cohorts). Consequently, the fields of rare diseases [3–7] and cancer [71–73]) are currently closest to the implementation of personalized medicine.

**Fig. 5:**
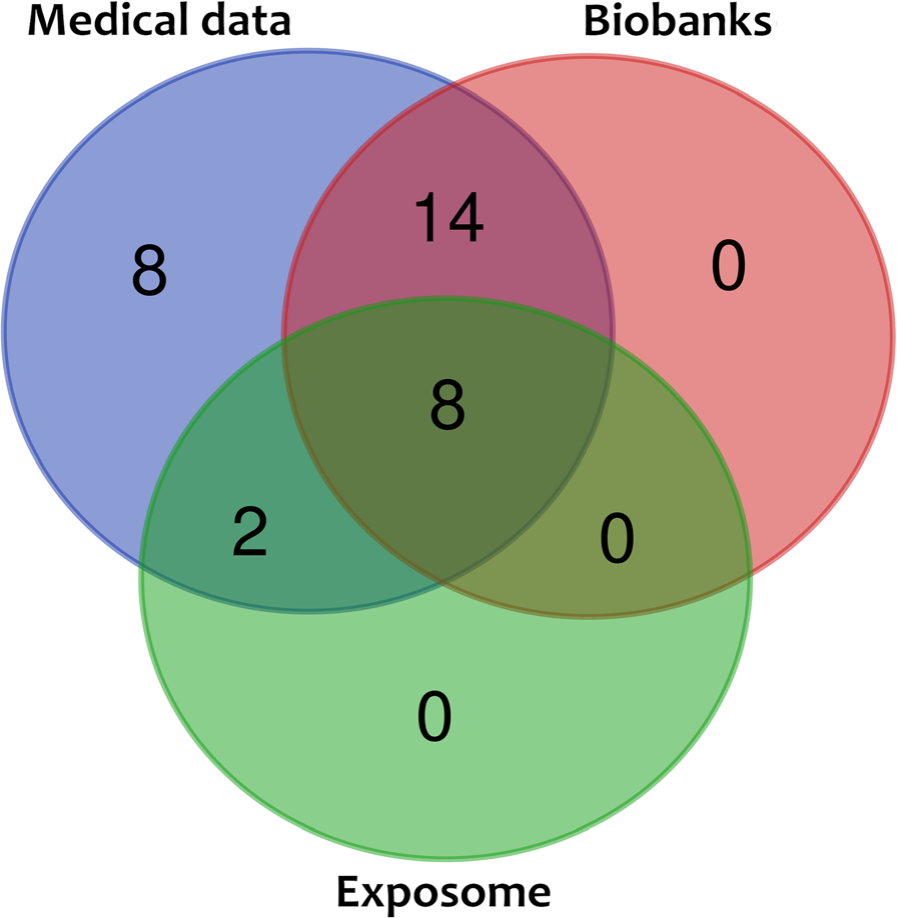
Overlap between 32 projects linking genomic data with biobanks, medical and non-medical data

Additionally, population genomics has helped us to better understand complex diseases and traits. Indeed, many national projects, for example, Finland and Estonia, report they will link their genomic effort with existing national prevention and intervention health programs in order to maximise their positive impact [29, 74]. The currently active projects have multiple, overlapping aims (Fig. 5) and the different strategies in which they intend to achieve them will be further discussed below.

#### Determining normal genomic variation

The most common goal among the national genomic projects was to determine normal genomic variation through the sequencing of presumably healthy population cohorts. This is not surprising, as determining the genomic variability/genomic background in the general population is necessary for a polygenic risk assessment approach to various complex and multifactorial human diseases. Furthermore, knowledge of the normal population-specific genomic variation helps improve the diagnostic yield of WES and whole-genome sequencing, showing a research return on investment in a short time-frame. Defining health in the context of genomic testing can be challenging, especially in the case of non-penetrant mutations, late-onset disorders, etc. Therefore, most national projects approached this challenge by either creating demographic cohorts and linking them with medical data or specific exclusion criteria, or by specifically identifying healthy individuals (healthy parents from trio testing in rare diseases, longitudinal health-tracking cohorts from previous studies, etc.), as is further discussed under the ‘Number and age structure of the included subjects’ section. Nine projects designed their normal genomic variability cohorts based on ethnicity data (Supplement Table 1). This approach is preferable, especially in case of large countries with many ethnic groups, or countries with considerable migration (both historic and present).

#### Determining pathological genomic variation

Genomic projects traditionally focused predominantly on rare disease and cancer cohorts. This approach has proved successful, and personalised medicine has begun in both of these fields [3–7, 72, 73].

The 29 countries with clinical cohorts approach this issue in various ways (Supplement Table 1). France, for example, plans to sequence over 235,000 genomes of at least 48 cohorts with clearly defined genetic conditions [30], UK plans to sequence approximately 100,000 patient genomes (rare diseases programme, which includes over 190 rare diseases, and cancer programme) [37], Australia and Hong Kong aim to sequence 20,000 patients each (18 rare disease and cancer flagship projects) [41, 66], while Thailand [39], New Zealand [21] and Slovenia [54] each plan a few hundred patients from rare disease and cancers cohorts. In the remainder of the countries that have clearly indicated the diseases included in their clinical cohorts (e.g. Ireland: rare disorders and 10 chronic conditions), the numbers of included patients remain to be finalized.

As can be seen from the results, most larger projects have made provisions to sequence complex clinical cohorts. Interestingly, as far as the composition of their cohorts can be analysed from the data provided on the websites, it is apparent how other factors, such as funding clearly influence clinical priority in genomics. Large, initially publicly funded initiatives such as that of UK and France [30, 37] have very complex clinical cohorts including over 190 rare diseases and cancer programme, in case of the former, and 48 conditions in case of the latter. On the other hand, privately funded projects will focus primarily on conditions where the biggest return on investment can most reasonably be expected (e.g. Ireland project aims to focus on Alzheimer’s disease, asthma, inflammatory bowel disease, multiple sclerosis, diabetes, nonalcoholic liver diseases, inflammatory skin conditions, ankylosing spondylitis, etc.).

#### Infrastructure

Infrastructural goals include the most heterogeneous aims, ranging from establishing new and linking existing sequencing facilities (e.g. France), improving computing/analysing capacities (e.g. Brazil, Portugal), establishing standards for analysis (e.g. Slovenia), data management (e.g. Finland, Switzerland), sharing and platform building (e.g. Estonia), education of medical personnel and incorporation of sequencing technology/diagnostics into existing health-care structures (e.g. Finland, France). This is not surprising, as the national genomic projects defined their infrastructural goals depending on their respective general situation regarding genomic sequencing and health-care systems. The most shared infrastructural goals were data management (79%, 19/24), standards of analyses (71%, 17/24), and interestingly, the goal of education-which was defined as the aim of half (54%, 13/24) of countries with infrastructural projects; however, these include major projects such as that of Australia, UK and Finland (with excellent existing health-care informatics infrastructure). Interestingly, a few projects (e.g. Slovenia) defined the goals of education, standards of analyses, and data management independently of infrastructure-highlighting the differences in the definition of the concept of ‘infrastructure’ itself. Furthermore, 68% of projects (28/41) reported their intention to unify or establish standards for analysis and/or make provisions for appropriate data management, which is not surprising as these two factors are crucial features for the establishment of personalized and precision medicine [75].

#### Personalised and precision medicine

The majority of the national genomic projects aspire to integrate personalized medicine with their existing healthcare infrastructure. However, only a third (37%, 15/41) of the projects have thus far proposed specific strategies for the implementation of personalised medicine. Preparing the ground for implementing personalised and precision medicine is a complex endeavour as it cannot precede the achievement of other important goals, such as identifying and cataloguing local normal genomic variability, the existence of adequate sequencing and informatics infrastructure, data security, clear ethical guidelines for reporting and interventions, education of medical professionals and health-care system integration.

Indeed, in most countries with tangible plans of implementing personalized medicine, this aim overlaps with all three other major aims: determining normal and pathological genomic variability, and infrastructural aims (Fig. 5). Therefore, the countries pursuing more aims are most likely to implement personalised medicine in the foreseeable future. For example, Finland, with its well-established medical data infrastructure, is in a good position to undertake the personal genomics challenge posed by complex diseases [29]. Additionally, several countries, such as Japan [76], report they will use their genomic data for drug discovery/precision therapy and have planned their cohorts accordingly.

### Number and age structure of the included subjects

The 34 national projects reporting the number of subjects to be included showed high heterogeneity, ranging from a hundred subjects to over a million individuals (Table 1).

In terms of sequenced genome numbers per country population, only four countries plan to sequence more than 1% of their respective population, while the majority of projects plan to sequence less than 0.2% of their population (Table 1). Five countries defined the age structure of their healthy participants (Supplement Table 1). For example, the Estonian national genomic project aims to analyse 32.5% of the country’s population and reports the plan to link this information with the national biobank, medical data and non-medical exposome data. Furthermore, in Estonia, the subjects for sequencing will be chosen to reflect the age structure in the country [74]. Similarly, the Latvian genome project will analyse healthy adult individuals included in their genetic biobank [32]. In the Czech Republic, approximately half of the healthy subjects included in the population cohort reflect the general population, whereas the other half is composed of healthy subjects above the age of 70 years [49]. Likewise, in Malta, a senior citizen cohort will be used to determine the normal genomic variation background [45]. Additionally, despite the relatively low number of planned genomic analyses, Brazil has an excellent population cohort from which to choose those to be sequenced. The Brazilian public servant cohort—ELSA (Longitudinal Study of Adult Health) has tracked the health of public servants aged 35–74 years and the factors associating with complex diseases since 2008 [77]. As discussed under normal genomic variability section, nine projects designed their cohorts based on ethnicity data (Supplement Table 1), which should be the preferred approach in case of countries with considerable migration and/or many different ethnic groups.

### Funding

Similarly, to the reported range in the number of subjects, the funding amounts vary greatly from less than a million USD to 9.2 billion USD. Approximately half (49%, 20/41) of ongoing projects have public funding, which is not a surprise given a high initial investment and unlikely short-term return on the research performed. The remaining projects have either mixed (44%, 18/41) or fully private (7%, 3/41) funding. The private partners of the mixed public-private funded projects are either sequencing companies such as Illumina, Macrogen, BGI, and insurance companies, research and pharmaceutical companies, universities or a combination of several such partners. Additionally, several projects report they will collaborate and/or share data with private companies in the future (Supplement table 2 and 3).

An interesting comparison can be made between the approach to the selection of the clinical cohorts based on the type of funding, which is mixed (initially public) in the case of France and private in the case of Ireland. While the clinical cohorts of the initially publicly funded project were chosen based on their potential public-health impact as well as scientific rationale, the cohorts included in the fully privately funded project reflect the conditions where the biggest return on investment can most reasonably be expected: Alzheimer’s disease, asthma, inflammatory bowel disease, multiple sclerosis, diabetes, liver disease, inflammatory skin conditions, ankylosing spondylitis, and non-radiographic axial spondyloarthritis, and rare disorders. Similarly, the privately funded Qatar national project aims to analyse 100,000 individual genomes (3.6% of the population) and so far reports only clinical cohorts consisting of cardiovascular disease, diabetes, neurological disease and cancer.

Private funding of genomic research represents several challenges that have been reviewed previously [78]; however, few countries possess adequate resources to be able to pursue genomics from research to full implementation of personalised medicine without outside involvement.

As a possible solution to these challenges, several countries plan to establish designated agencies that will act as gatekeepers between the public and private conflict(s) of interest (e.g. data-security versus profit), in order to enable interested private parties to join the project and get involved in generating added value (design of novel drugs, treatments, data-mining), while maintaining public control of the data itself, as far (and as long), as possible.

### Data sharing goals and methods

Data sharing in the context of genomic projects concerns ethics and legal issues, identifying stakeholders, as well as technical aspects and security of the data itself. The ethical and legal issues depend on each national project as well as the projects' funding (public vs. private). The interested parties have been identified by several projects (please see Supplement Table 3 for detailed information) as the patient/healthy participants, referring physicians, the general public, researchers and research organizations, private corporations (such as pharmaceutical and insurance companies) and international organizations. The different stakeholders can access various levels of data either through fully public databases containing de-identified information or by formal request to the particular national ethical committee. Regarding the technical solutions for data sharing, some projects have already provided dissemination platforms, data access per request, or a synopsis of their results, whereas the remainder have announced their plans to do so (Supplement Table 3). The projects with significant funding, such as that of USA [79], China [80], UK [12], Australia [81], Japan [82] and Switzerland [82] as well as smaller projects such as Brazil [83], Latvia [84] and Saudi Arabia [85], have designed database platforms with various levels of access (for the interested public, academia and researchers), whereas probably due to significantly lesser financial input, the majority of projects created public databases with anonymized or pooled genetic data [42, 67, 86–90].

Additionally, five countries, Denmark, Estonia, France, Latvia and Qatar either have or plan to make available both data and the collected DNA, per request and pending the approval of their Ethical Committee (Supplement Table 3).

### Linkage with biobanks, medical and non-medical data

Unsurprisingly, most of the ongoing projects aim to link the sequencing data with other medical data (78%, 32/41), either as part of their reported clinical cohorts, existing medical infrastructure or collected de novo. Furthermore, likely because of the high costs associated with such operations and their maintenance, roughly half of these projects (54%, 22/41) will integrate the results of the sequencing experiments with the existing biobanks or will create such biobanks as part of the project (Table 2).

The projects in the best position to achieve this goal are those of relatively small countries with a public healthcare system and well-established biobanks, such as Finland and Estonia. Estonia’s biobank includes close to 200,000 participants with information on their medical history, current health status and medications, in addition to anthropometric measurements and blood aliquots. In the case of Finland, the genome database will be linked with the existing National Health Data Repository (Kanta), which is already integrated into the public healthcare system. Pilot projects supporting the utilization of genomic data in Finnish healthcare, such as the GeneRISK study, that aim to analyse how information about risk-factors influences lifestyle changes and acts to prevent disease, are already underway [29].

Linkage with non-medical data, reported by 24% of projects (10/41), was less clearly defined as the exposome is both difficult to define and measure, and requires a significant investment in terms of the effort to collect and perform analyses. Current strategies examining the human exposome, that is the totality of lifetime-exposure, include many different factors (lifestyle, environment, microbiome, pollutants (sound, chemical), stress, etc.) and remain far from standardized. However, despite the fact that many issues remain before this field can be standardised, our efforts should strive to enable the linkage of data between studies in the future [91–94], and it is foreseeable that the exposome-genome paradigm will strengthen the application of precision medicine as these fields progress [95].

### Challenges and future directions

Our aim was to provide an overview of available information on active national genome projects worldwide, in order to aid the design of such projects and usefulness of their results. We showed that despite the obvious, and substantial, diversity of the 41 ongoing projects, their overarching efforts aim to overcome the existing barriers to obtaining data, its integration, and the translation of this knowledge into personalised medicine. The challenges for this ambitious aim are many, such as addressing data security, privacy issues, inconsistency in data generation and analysis, issues with data sharing resources (both technical and ethical), incompatible data models and terminology, etc. The projects we have reviewed approach these issues in different ways, although some have already recognised the need to standardise their efforts in order to enable an interoperable framework of responsible data sharing.

Open science initiatives, such as the Global Alliance for Genomics and Health (GA4GH) [96], have been established to address the need for common standards and approaches to using genomic and related data. Their standards have so far been adopted by more than 40 leading genomics institutions as well as several of the projects described in this report, such as ‘All of Us’ USA, Genomics England, Australian Genomics, and Slovenia, to name a few, and will hopefully be even more widely adopted by such projects in the future.

Additionally, we would like to suggest that in isolation, genomic data represents only a part of the larger effort needed for implementing personalised and precision medicine, and as more and more genomes are included, the need for supporting medical and non-medical data (exposomics, integratomics, etc.) has become more and more apparent. Therefore, we would like to suggest that it is preferable for project designers to make provisions for the systematic inclusion of additional, medical and environmental/exposure data that will enable better genomic data curation and interpretation. It is foreseeable that in this aspect, open science initiatives will once again prove helpful in enabling frameworks and standards for successful data integration.

### Limitations of the study

The study faces several limitations. Firstly, the information obtained by the authors is based on what was provided on the web sites of individual national projects in the English language. As individual projects’ websites do not need to adhere to standards as strict as those of scientific publishing, this prevented us from fully following all of the principles outlined as part of the PRISMA approach to systematic reviews and meta-analyses [19]. We would also like to recognize that our analysis may not reflect the full or final scope of the individual projects.

Secondly, all information we have attempted to gather was not available in case of all projects or was yet to be determined. The final scope and results of several projects will depend on the results of their many pilots and supporting/preparatory measures. Therefore, we would like to point out that perhaps not all aims may be achieved to the extent envisioned initially and that possibly additional features will be added to many of the projects at a later date.

In case of determining both healthy and pathological genomic variation, the recruitment of cohorts is an ongoing process that may result in changes to the original proposal, and new technological solutions, ethical standards and the results of international efforts (such as the European ‘1+ Million Genomes’ Initiative) may and probably will act to (re)shape the projects in the future.

Finally, this study was partly conducted during the COVID-19 global pandemic, which may influence the national genomic projects in unforeseeable ways.

## Conclusions

In conclusion, this systematic review demonstrated considerable diversity among the 41 currently ongoing national genomic projects. The overview of the existing designs will hopefully inform national initiatives in designing new genomic projects and contribute to standardisation and international collaboration, thus enabling the individual projects to better contribute to the global development of genomics and personalized medicine.

## Supplementary Information

**Additional file 1: Table S1**. Identified national genomic projects and categories analyzed in ongoing national genomic projects.

**Additional file 2: Table S2**. Funding details of national genomic projects.

**Additional file 3: Table S3**. Data sharing solutions of national genomic projects.

## Data Availability

All data generated or analysed during this study are included in this published article [and its supplementary information files].

## Abbreviations

WES: Whole exome sequencing
